# The Emergence of Synaesthesia in a Neuronal Network Model via Changes in Perceptual Sensitivity and Plasticity

**DOI:** 10.1371/journal.pcbi.1004959

**Published:** 2016-07-08

**Authors:** Oren Shriki, Yaniv Sadeh, Jamie Ward

**Affiliations:** 1 Department of Cognitive and Brain Sciences, Ben-Gurion University, Beer-Sheva, Israel; 2 Department of Computer Science, Ben-Gurion University, Beer-Sheva, Israel; 3 Zlotowski Center for Neuroscience, Ben Gurion University, Beer-Sheva, Israel; 4 Israel Arts and Science Academy, Jerusalem, Israel; 5 School of Psychology, University of Sussex, Brighton, United Kingdom; 6 Sackler Centre for Consciousness Science, University of Sussex, Brighton, United Kingdom; Duke University, UNITED STATES

## Abstract

Synaesthesia is an unusual perceptual experience in which an inducer stimulus triggers a percept in a different domain in addition to its own. To explore the conditions under which synaesthesia evolves, we studied a neuronal network model that represents two recurrently connected neural systems. The interactions in the network evolve according to learning rules that optimize sensory sensitivity. We demonstrate several scenarios, such as sensory deprivation or heightened plasticity, under which synaesthesia can evolve even though the inputs to the two systems are statistically independent and the initial cross-talk interactions are zero. Sensory deprivation is the known causal mechanism for acquired synaesthesia and increased plasticity is implicated in developmental synaesthesia. The model unifies different causes of synaesthesia within a single theoretical framework and repositions synaesthesia not as some quirk of aberrant connectivity, but rather as a functional brain state that can emerge as a consequence of optimising sensory information processing.

## Author Summary

Synaesthesia is a remarkable form of altered perception, where one attribute of a stimulus (e.g. sound) leads to the conscious experience of an additional attribute (often colour). Despite being known about for 200 years, there is no commonly agreed upon model for how and why synaesthesia emerges. This study presents a new model of synaesthesia based on computational principles that accounts for the emergence of different types of synaesthesia (acquired and developmental) as well as many of its key characteristics. The model describes how two independent neuronal systems can evolve to interact with one another even though their inputs are statistically uncorrelated. Specifically, synaesthesia arises as a result of instability in the learning process that shapes the network, which can be caused by heightened plasticity or due to sensory deprivation of one of the systems. The model unifies different aspects of synaesthesia and generates novel insights and predictions.

## Introduction

Synaesthesia is a remarkable form of altered perception. One attribute of a stimulus (e.g. its sound, shape or meaning) may inevitably lead to the conscious experience of an additional attribute (often colour). For example, the word “Phillip” may taste of sour oranges, the grapheme A may be bright red, and a C# note on the violin may be a brown fuzzy line extending from left to right in the lower left part of space [1]. Although the precise definition of synaesthesia remains open to debate [2], there are a number of generally agreed upon characteristics. The first is that synaesthesia is elicited by particular stimuli (unlike hallucinations which may occur spontaneously). The stimulus that elicits the synaesthesia is termed the inducer and the synaesthetic experience itself is the concurrent [3]. A second characteristic is that the experience is automatic. That is, the inducer inevitably leads to the concurrent experience. Finally, synaesthetic concurrents are (from a first-person perspective) described as being percept-like. In corroboration of this, functional imaging studies have often found activity within perceptual regions (e.g. colour-sensitive regions) when synaesthesia is experienced [4].

### Existing Accounts of the Causes and Mechanisms of Synaesthesia

A broad distinction made in the synaesthesia literature is between acquired and developmental forms and it is presently unclear whether a single model or mechanism can account for them both.

Developmental forms of synaesthesia have no known triggering event. The typical explanation is that genetic differences in these individuals give rise to structural and functional differences in their brains [5]. Genetic differences linked to synaesthesia have been identified and synaesthesia is known to run in families [e.g. 6]. However, the exact synaesthetic associations themselves do not appear to be inherited, despite being stable within individuals. Thus, a mother may perceive ‘A’ as red and her daughter may perceive it as blue [7]. One of the earlier ways of describing synaesthesia is in terms of a breakdown in modularity [8]. In effect, a given brain region (e.g. that responsible for colour perception) responds to multiple inputs in synaesthetes but not others (e.g. responding to sounds or achromatic letters as well as colours). The evidence from functional imaging generally supports this idea [4].

Ramachandran and Hubbard [9] suggest that adjacent regions of cortex may be particularly predisposed to pair as synaesthetic inducers and concurrents in developmental synaesthesia. This may explain why combinations such as grapheme-colour synaesthesia are particularly prevalent [10]; i.e. because of anatomical proximity within the visual ventral stream of grapheme recognition and colour perception. Computational models in general have suggested that a high degree of local clustering is an optimal solution for cortico-cortical connectivity [11].

Although some cases of developmental synaesthesia appear to have derived their associations from, say, alphabet books/blocks this is not the norm [12, 13]. Similarly, most people exposed to coloured alphabets do not develop synaesthesia. Moreover, for some synaesthetes the spoken or written word “red” may even be synaesthetically blue, or some other colour [14]. As such, associative learning does not seem a plausible general mechanism. However, the mapping between inducers and concurrents is not random. Monotonic mappings have been reported in a variety of types of synaesthesia: increasing pitch is associated with increased luminance in auditory-visual synaesthesia [15]; increased weight is associated with decreased luminance in tactile-visual synaesthesia [16]; and increasing numerosity of digits is linked to decreasing saturation and luminance in number-colour synaesthesia [17]. In the case of letter-colour synaesthesia, there appear to be multiple influences: colours depend on the shapes of letters and their frequency in the alphabet [18]. Synaesthesia tends to be unidirectional such that, for example, a sound may trigger a colour but a colour doesn’t trigger a sound. However, there is some evidence that bidirectionality may occur implicitly (e.g. a colour may speed up detection of a subsequent grapheme), and a few cases in which it has been documented to occur explicitly [19]. When bidirectional synaesthesia is present it need not be symmetrical; for instance, a given sound may trigger a red colour, but seeing a red colour triggers a very different sound [20].

With regards to acquired synaesthesia, there is a known triggering event that leads to the onset of synaesthesia. Synaesthesia can be acquired in two different ways—as a result of sensory impairments [e.g. blindness 21] or as a result of taking certain drugs such as LSD (lysergic acid diethylamide[22]). The latter tends to be temporary and occurs quickly (minutes, hours), whereas the former can occur either quickly (days) or slowly (months or years) and lasts for long or indefinite periods. Superficially, acquired synaesthesia appears to have somewhat different characteristics from developmental forms of synaesthesia. The nature of the inducer tends to be a sensory stimulus: there are no instances on record of acquired grapheme-colour synaesthesia, for example. This faster acting mechanism is consistent with unmasking (i.e. removal of inhibition) of pathways that are already established or enhancement of existing excitatory interactions. For instance, after blindfolding for a few days the ‘visual’ cortex responds to inputs from touch and audition [23]. Although this is not strictly synaesthesia, it represents an example of an inducer triggering a concurrent in neurophysiological terms if not in terms of perceptual experience. In addition to changes in inhibition/excitation, there may be slower-acting structural changes [e.g. synaptogenesis along multi-sensory pathways 24] that lead to acquired synaesthesia and explains why the onset of synaesthesia can occur up to a year after sensory loss.

### Computational Models of Synaesthesia

There is only one known computational model of synaesthesia [25]. This model is based on a self-organising Kohonen network and was established to account for one very specific type of synaesthesia: a tendency of some people to experience the sequence of numbers in a spatial configuration. The approach taken in the present study is very different in that it aims to offer a general account of the kinds of scenarios in which synaesthesia might evolve from a neural network and is not seeking to model any particular variety of synaesthesia.

**Fig 1:**
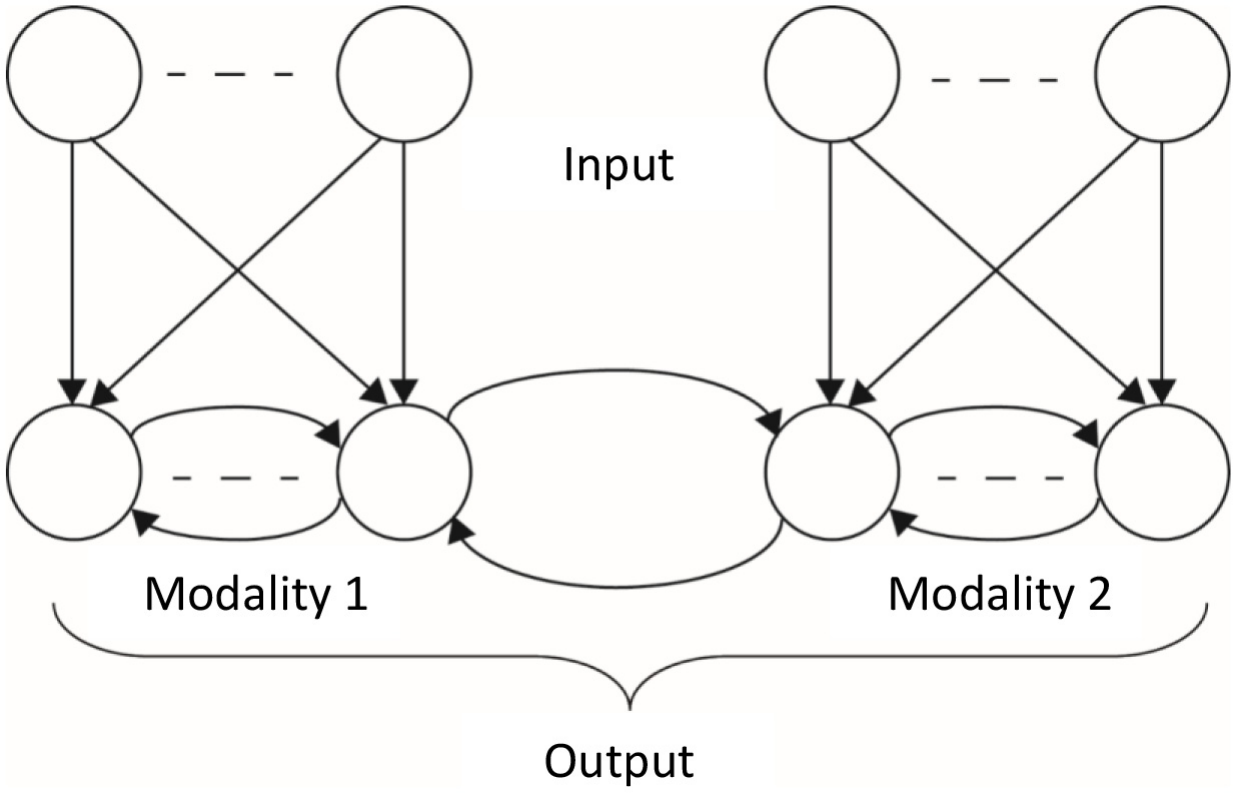
Architecture of a general network model for studying synaesthesia. The network consists of two interacting modalities. Each modality has a set of input neurons and a set of output neurons. There are feedforward connections within each modality but not between them. There are recurrent connections among all output neurons. The subset of recurrent connections that connect the two modalities are referred to as cross-talk interactions. The goal of the network is to optimize the representation of the combined input from both sets of input neurons, by the neurons at the combined output layer.

The basic architecture of the model below contains two sets of units that can be construed as different modalities (or, rather, features within a modality). The two different sets of input neurons connect to additional layers of output neurons (Fig 1). The neurons in each output layer are connected by recurrent connections and additional recurrent connections connect the two output layers with one another.

In order for synaesthesia to evolve in the first place it would require connections to already be in place between the two modalities, although not necessarily functional. This is developmentally plausible [26]. In our model the difference between synaesthetes and non-synaesthetes lies in whether these connections become functional as a result of the learning process. The presence of synaesthesia is thus operationalised as stable non-zero cross-talk connections between modalities 1 and 2 (or vice versa), together with the observation that stimulating one set of inputs activates both modalities (i.e. 1→1+2 and/or 2→1+2).

The evolution of the recurrent connections in the network, both internal and cross-talk, is governed by learning rules that optimise the information representation of the external inputs into the modalities [27, 28]. More specifically, the quality of the representation is measured by the *mutual information* [29] between the input to the network and the neuronal output. Here the input corresponds to the total input to both modalities and similarly the output corresponds to the total output of both modalities after reaching steady state. In our context, the mutual information reflects the ability of the network to discriminate between two similar inputs or, in other words, its sensitivity to changes in the external inputs.

In the beginning of the learning process, the cross-talk connections are set to near zero. During learning, the network is presented with input samples of certain statistical characteristics. A major question relates to the role of statistical correlations between the inputs to both modalities. If the inputs are statistically correlated, it is not surprising that cross-talk connections will evolve. From a computational point of view the network can take advantage of these correlations and improve the quality of the representation. However, it seems that in most real-world cases no such correlation underlies synaesthesia. Thus, we try to examine the conditions under which synaesthesia develops despite the fact that there are no correlations between the inputs. In our network model, when the inputs to the two modalities are uncorrelated, typically no cross-talk connections evolve. However, as we show in the following sections, under certain conditions they develop and synaesthesia emerges.

## Results

### Evolution of Cross-Talk in a Simple Network Model with Two Interconnected Units

**Fig 2:**
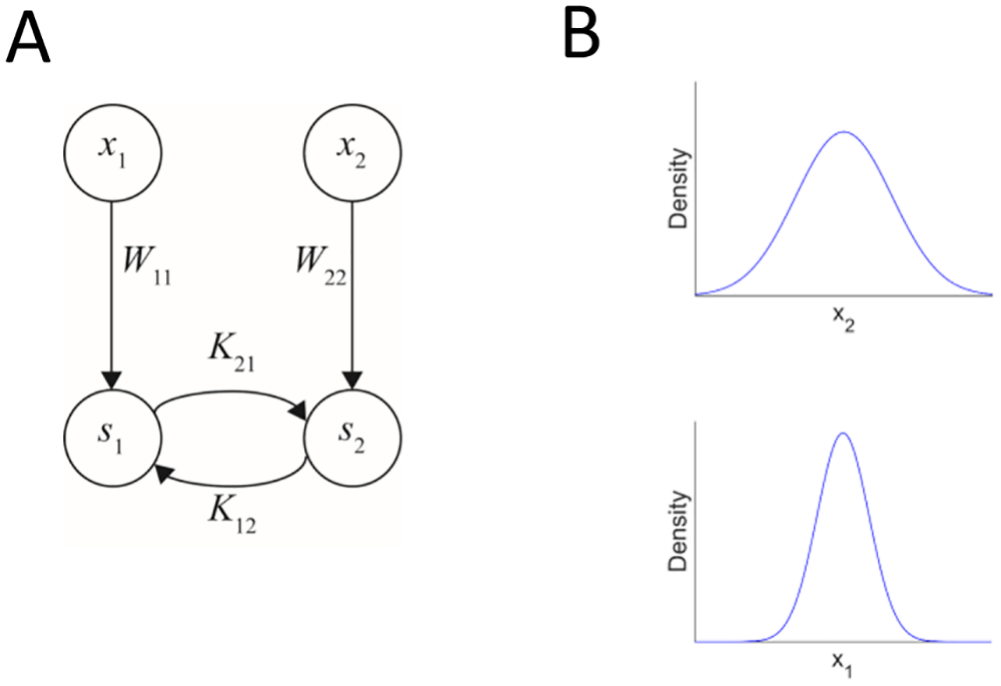
A simple network architecture to study the conditions for evolution of cross-talk interactions. (A) The network contains four neurons, one input neuron and one output neuron in each modality. The feedforward connections are denoted by W_11_ and W_22_ and the recurrent cross-talk connections are denoted by K_12_ (from neuron 2 to 1) and K_21_ (from neuron 1 to 2). In this simple case, there are no internal recurrent connections within each modality, only between them. (B) The inputs were drawn from two independent Gaussian distributions with zero mean. We analysed the effect of the variances of the two Gaussian distributions on the evolution of cross-talk connections.

**Fig 3:**
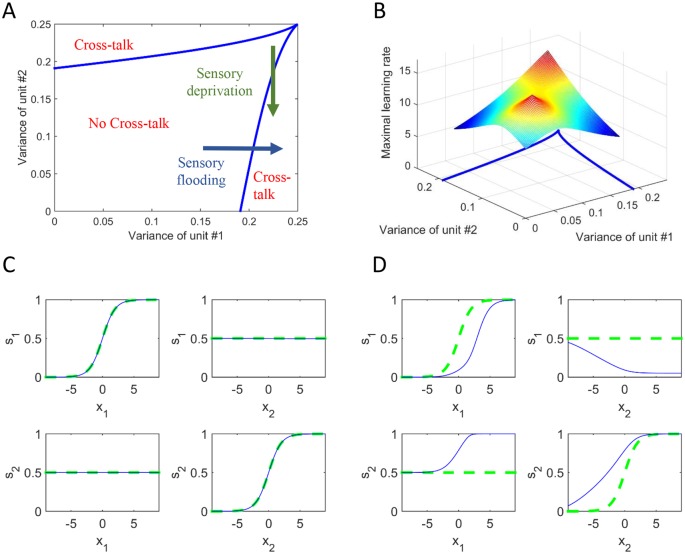
Conditions on input statistics and network plasticity for the evolution of cross-talk interactions. A phase-diagram of stability as a function of output variances based on mathematical analysis (A) and additionally including the critical learning rate (B). There is a regime of stability where no cross-talk develops. Outside this regime, cross-talk connections evolve. The scenarios for losing stability are analogous to ***sensory deprivation*** or ***sensory flooding***. C and D show the input and output activity for two specific scenarios depicting no cross-talk (C) and cross-talk (D). In all cases, one input was changed while the other was kept at 0. Thus, the panels show the response of each unit either to its direct input or to input to the other unit. The green dotted curve represents the state of the model before the beginning of the learning process and the blue solid curve represents the state after the learning process. In D, the solid blue curve represents the responses after the emergence of synaesthesia. In this particular case, the interactions evolved to: K_12_ = -2.91 (2 inhibits 1), and K_21_ = 14.34 (1 excites 2).

We first analyze a network where each modality contains a single input neuron and a single output neuron (Fig 2). The simplicity of this network model makes it amenable to analytical investigation in addition to computational simulations. The input and output neurons in each modality are connected in a feed-forward manner. The input to each modality is taken to be normally distributed with zero mean, and the two one-dimensional distributions are statistically independent. There are additional recurrent (*cross-talk*) connections between the two output units. Synaesthesia evolves when the cross-talk connections between the two units increase and become functional. In order to determine the evolution of synaesthesia, we first identify the conditions under which zero cross-talk connectivity (K_12_ = K_21_ = 0) is a fixed-point of the learning dynamics, and then look for the conditions under which this fixed-point becomes unstable. In other words, the question is what will happen to a small perturbation to the connections. If both connections go back to zero, the no-cross-talk state is a stable state. The interesting case is when this state becomes unstable and the cross-talk connections develop. The information maximization learning rules for the connections K_12_ and K_21_ form a set of two coupled nonlinear equations. We linearized these equations around the point K_12_ = K_21_ = 0 and explored the discrete time dynamics by analysing the corresponding eigenvalues. The details of the investigation appear in the Supporting Information and the Results are summarised in Fig 3.

We first analysed how the stability of the fixed-point depends on the variances of the two output neurons. These variances are determined by the variances of the Gaussian distributions at the input. The higher the input variance, the higher the output variance, but due to the bounded non-linearity of the output neurons, the output variance is constrained to be between 0 and 0.25 (see Supporting Information). Fig 3A shows the phase diagram of the stability as a function of the two output variances. For pairs of variances in the central region, the no-cross-talk state is stable. Outside this region cross-talk connections evolve (i.e. synaesthesia occurs). There are various scenarios in which a network can be driven outside the no-cross-talk region. For instance, consider a situation in which the variance of the second unit is decreased (shown by the green arrow). This situation is analogous to *sensory deprivation* at the second unit. At the same point the network develops cross-talk connections from the non-deprived unit to the deprived unit, which increase the output variance at the deprived unit. Similarly, cross-talk connections evolve when the variance of the first unit is increased (shown by the blue arrow). This situation is analogous to *sensory flooding* at the first unit.

Fig 3B shows the same phase diagram together with a surface which describes the critical learning rate, *η*_*critical*_, as a function of the variances. Above this surface, synaesthesia appears (although the statistical variances alone give a "normal" state, without synaesthesia). This reflects instability of the learning dynamics due to the high plasticity. The interpretation is that people with high synaptic plasticity are more likely to develop synaesthesia. It cannot be seen in the graph (in order to have a satisfying resolution for the z-axis), but when both variances approach 0.25, the critical learning rate approaches infinity. This means that close to these variance values and in the main regime (of no cross-talk), the learning rate must be very large to result in cross-talk, or synaesthesia. Fig 3C and 3D represent two specific examples of end points within this model space. Fig 3C represents the more typical scenario of no cross-talk such that s1 is sensitive to inputs from x1 alone and s2 is sensitive to inputs from x2 alone. Fig 3D represents an example of the state of the model after the evolution of cross-talk under a sensory flooding scenario. In this model, s2 is activated by inputs from both x2 and x1 (i.e. a case of modality 1→ modality 2 synaesthesia). Note also how s2 has become more sensitive to its own inputs; that is, synaesthesia has increased unimodal sensitivity within the concurrent modality (modality 2). By contrast, the cross-modal inputs from modality 2 to modality 1 are negative (inhibitory); i.e. the synaesthesia is not bidirectional.

**Fig 4:**
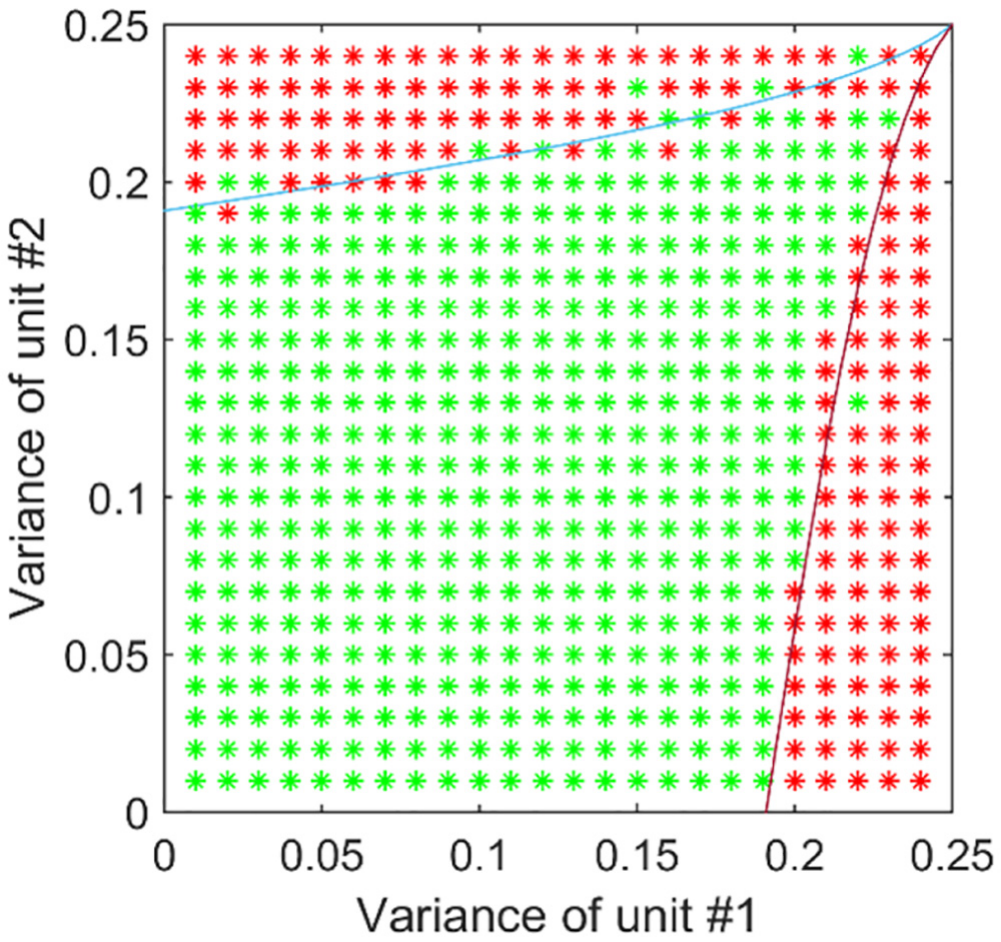
Simulation results for varying input statistics on the evolution of cross-talk interactions. Green asterisks represent an absence of cross-talk after a long time and red asterisks represent the presence of cross-talk. The results are in close agreement with the mathematical analysis (shown with the curved lines). Close to this borderline, the dynamics are very slow and the simulation may terminate before reaching the predicted solution. Differences in the outcome could also relate to the different initial conditions of the model (which were selected at random).

We next verified that results of the analytical investigation using numerical simulations of the corresponding network. The input to each modality was random and normally distributed. The range of variances was sampled in a resolution of about 0.01, and the total amount of simulations was 729 (27x27). In each simulation the learning process was run with a different pair of variances (of both units). The initial values for the cross-talk connections were randomly chosen in a ring around the origin (K_12_ = K_21_ = 0). In this case, we checked whether the network converged back to the no cross-talk state or diverged. The results (Fig 4) are consistent with the analytical calculations. The "leaking" of stable-points into the theoretical unstable-area and vice versa, and the asymmetry in respect to the major diagonal is the result of insufficient accuracy or not enough learning-steps in the simulation.

The simple model reveals a number of scenarios in which cross-talk may emerge between recurrently connected units, receiving different inputs, based on the principle of maximising the overall sensitivity of the network model. Decreased variance of the input is analogous to sensory deprivation, which is the known aetiology in most (if not all) cases of acquired synaesthesia. Sensory flooding (increased variance of one input) is another possible cause for synaesthesia. Synaesthetes also have better perceptual discrimination within the concurrent modality [30]. Interestingly, it has recently been found that autism, which is linked to sensory flooding, is also co-morbid with synaesthesia [e.g. 31]. Another finding is related to the learning rate. As the analysis shows, there is a critical value above which the network may develop synaesthesia. This prediction is consistent with the established fact that developmental synaesthesia usually occurs at an early age, when the brain is more plastic. It may also be related to the fact that developmental synaesthesia is linked to enhanced memory abilities [32].

### Evolution of Synaesthesia in a Network with a High-Dimensional Representation

The analysis of the simple model shows that the evolution of cross-talk connections occurs in several scenarios; namely sensory deprivation, sensory flooding and high plasticity. However, the existence of cross-talk in itself does not necessarily reflect synesthetic behavior, since synesthesia also requires a systematic mapping of inducers to concurrents. The aim of this section is to extend these findings in a more complex model containing a population of output units in each modality. In this scenario, each unit has the potential to represent a particular feature of the input and, therefore, it enables us to explore how features in one modality are mapped to features in the other modality. For instance, do monotonic mappings between features in different modalities emerge? Are they entirely idiosyncratic? Under which conditions do the mappings fluctuate or become stable? In synaesthesia, the mappings tend to be consistent within an individual. The mappings tend to differ across individuals but are not strictly random: for instance, synaesthetes tend to show monotonic relationships between pitch and luminance [15].

**Fig 5:**
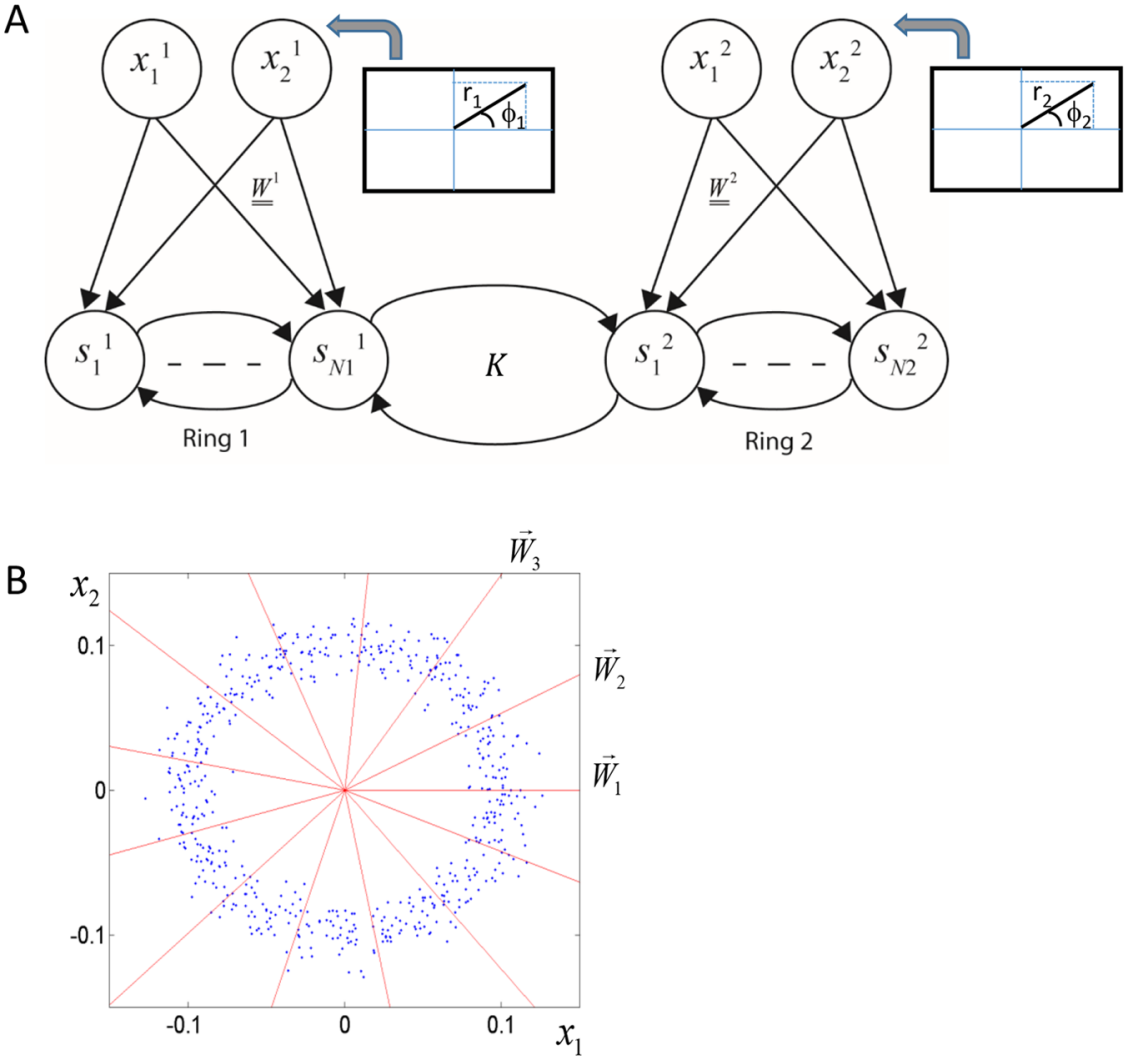
A network model for studying the evolution of synaesthetic mappings. A. Network architecture. The network is composed of two interacting modalities. Each modality receives a two-dimensional input characterized by an angle and a distance from the origin. This input is mapped into a high dimensional representation. There are recurrent connections among all the neurons in the output layer, namely within and between modalities. For clarity, only a few connections are shown. B. Feedforward connections and input distribution. The feedforward connections (red radial lines) are unit vectors with angles equally spaced from 0° to 360°. They are fixed throughout the learning. The input to each neuron is proportional to the projection of the input on the corresponding unit vector and has a cosine tuning around the corresponding angle, which represents its preferred feature. For clarity, the figure shows only a few lines, but in the numerical simulations we used 71 output neurons in each modality. The blue dots depict the input distribution to a single modality. The angles are uniformly distributed and the distance from the origin has a Gaussian distribution around a characteristic distance (0.1 in this example), which represents stimulus intensity.

In this model, the input to each modality is two-dimensional characterized by an angle and a distance from the origin (Fig 5). The angle, φ, represents a one-dimensional perceptual space (e.g. the pitch of a sound, the luminance of a colour) and the distance from the origin, r, represents intensity. The magnitudes, r, of the input samples were drawn from a normal distribution (with standard deviation proportional to the mean) and the angles were drawn from a uniform distribution (Fig 5B; blue dots). Altogether, there are four input-neurons, and the inputs to the two modalities are uncorrelated (Fig 5A). The network was presented with random inputs and the recurrent synaptic connections were updated according to the gradient-based learning rules.

The feed-forward connections were set to be unit vectors with different angles, θ_*i*_, which spanned all possible angles from 0° to 360° (Fig 5B; red radial lines). Thus, the weighted input to each neuron in the output layer is: *r* cos(θ_*i*_−φ). In this sense, the angle θ_*i*_ can be referred to as the *preferred angle* of the *i*'th neuron. An external stimulus at a given angle φ elicits a 'hill' of activity around the neuron with the closest preferred angle. Each modality in this model is similar to a visual hypercolumn, the basic functional unit of the primary visual cortex, which contains a representation of all possible orientations. Analysis of the behaviour of a single hypercolumn network model with these properties and the same information maximization approach appears in [28]. Here, we analyse the case of two coupled networks of this type.

In the simulations, we explored the effect of the mean input magnitude and of the plasticity (learning rate). In this model, like in the simple network, the cross-talk connections were initially set to near-zero. We assumed for simplicity that the level of plasticity is the same for all recurrent interactions in the network, and therefore used a single learning rate.

**Fig 6:**
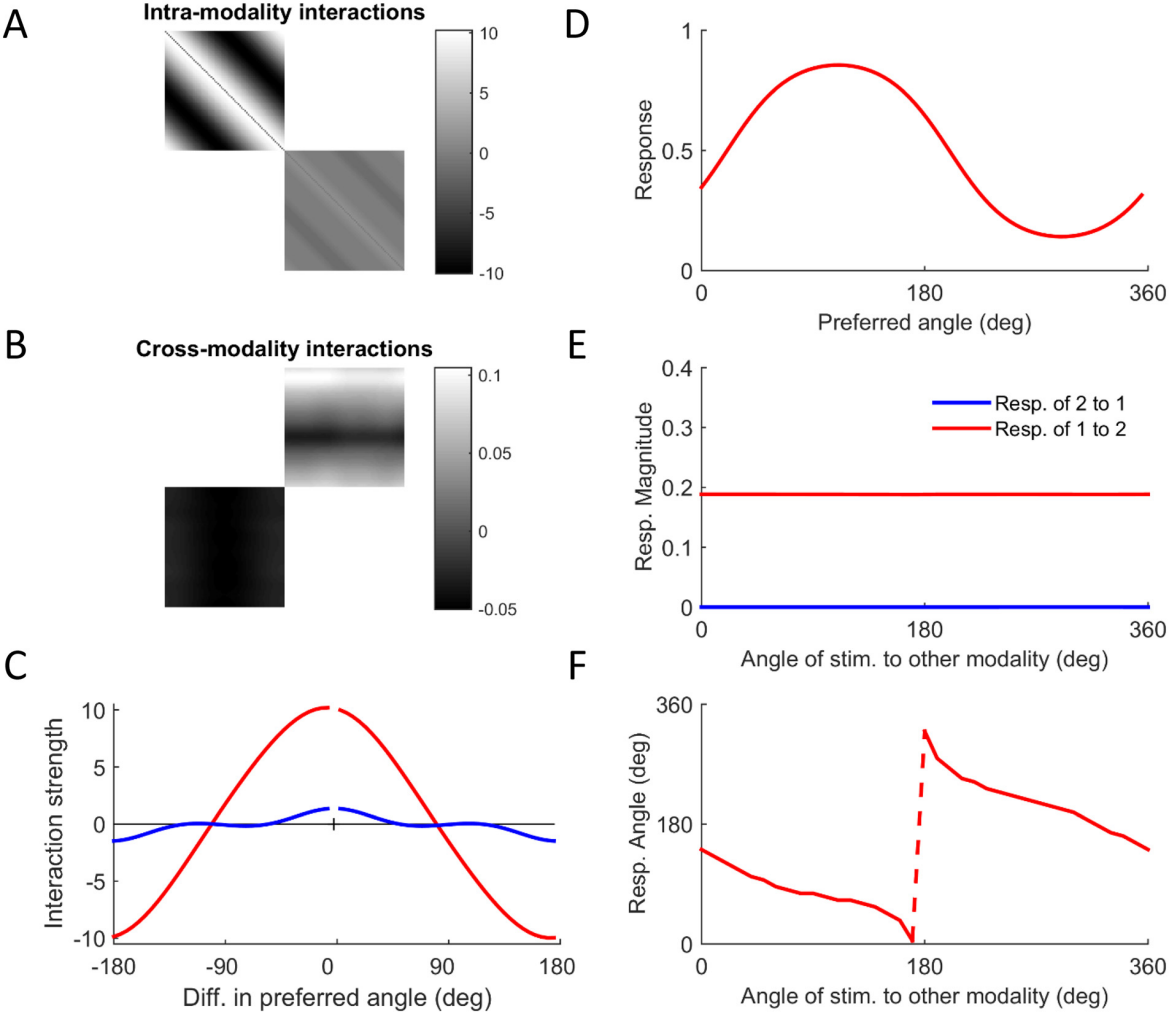
Evolution of synaesthetic mapping. The figure shows results from a simulation in which the network developed synaesthesia where modality 2 is the inducer and modality 1 is the concurrent. The input to modality 1 was *r*_1_ = 0.2 and the input to modality 2 was *r*_2_ = 2. A. Pattern of intra-modality interactions. Note that the higher contrast image (top left) reflects the greater sensitivity of modality 1 to its own inputs. B. Pattern of cross-modality interactions. C. Interaction profiles within modality 1 (red) and within modality 2 (blue). D. Response of modality 1 to single stimulation of modality 2 at an angle of 30 deg. E. Magnitude of population vectors shows the synaesthesia to be unidirectional. F. Synaesthetic mapping from stimulation of modality 2 to response of modality 1 showing a shifted monotonic relationship. (The sharp jump is due to the periodicity of the angle).

The network showed various types of behavior depending on the learning rates and input statistics. An example is shown in Fig 6. In this simulation, the characteristic magnitudes of the inputs were r_1_ = 0.2 and r_2_ = 2. This situation is analogous to sensory deprivation of modality 1. The recurrent interaction matrix has a block structure, where the diagonal blocks (Fig 6A) correspond to the interactions within each modality and the off-diagonal blocks (Fig 6B) correspond to the cross-talk interactions. The cross-talk interactions are much weaker compared to the interactions within each modality, as evident by the corresponding scale bars. The interactions within each modality are symmetric and they are excitatory for neurons with similar preferred angles and inhibitory for neurons with more distant preferred angles [28]. However, the strength of the interactions is much stronger in modality 1, the deprived one, reflecting stronger amplification of its direct inputs (Fig 6C). The cross-talk interactions from modality 2 to modality 1 are mainly excitatory, whereas the cross talk interactions from modality 1 to modality 2 are mainly inhibitory (Fig 6B), resembling the behaviour of the simple model (Fig 3D).

We also checked the existence of synesthetic behavior by directly stimulating one modality and testing the response of the other. Fig 6D shows the response of modality 1 to stimulation of modality 2 at an angle of 30°. A compact representation of the response is provided by the magnitude and angle of the *population vector* (Methods; [28]). The magnitude of the population vector of modality 1 in response to stimulation of modality 2 at different angles is finite (Fig 6E, red). In contrast, the magnitude of the population vector of modality 2 in response to stimulation of modality 1 is effectively zero (Fig 6E, blue). The angle of the population vector of modality 1 in response to stimulation of modality 2 shows a clear systematic mapping (Fig 6F). The fact that the mapping is phase-shifted and decreasing is not important since the values are arbitrary, but the fact that there is a monotonic relationship at all is not trivial (given that no such mapping was present in the input)

**Fig 7:**
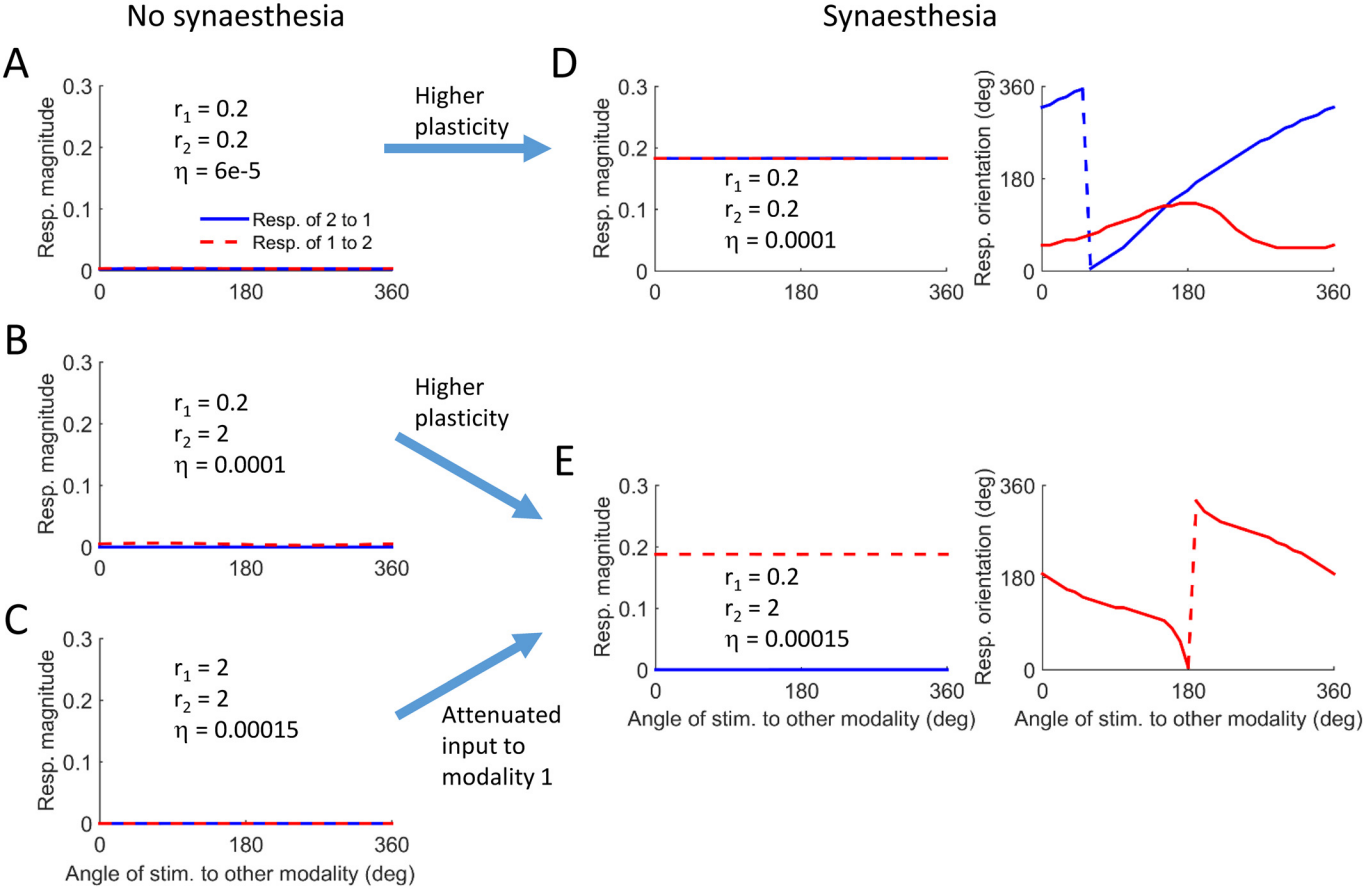
Different scenarios for the evolution of synaesthetic mapping in the model. A-C. Conditions on input statistics and learning rate for which no synaesthesia evolves. D-E. Conditions on input statistics and learning rate for which synaesthesia evolves. The arrows describe scenarios for the evolution of synaesthesia.

Fig 7 summarizes the results from 5 simulations and demonstrates the different scenarios that can lead to the evolution of synaesthesia. The values of the input magnitudes and the level of plasticity appear inside each panel. The first 3 simulations (Fig 7A–7C) describe conditions under which no synaesthesia evolved, resulting in population vectors with zero magnitude. The simulation in Fig 7D had the same input statistics as in Fig 7A (r_1_ = r_2_ = 0.2), but a slightly higher level of plasticity. The magnitude of the population vectors is finite in both directions, reflecting a bi-directional synaesthesia (Fig 7D, left panel). This is not surprising as there was complete symmetry between the two modalities in terms of the input statistics. Nevertheless, the mapping from modality 1 to modality 2 is monotonic, whereas the mapping in the opposite direction is non-monotonic (Fig 7D, right panel). This reflects some arbitrary symmetry breaking in the evolution of the cross-talk connection pattern. This may have been caused by small differences in the realization of the random inputs to the modalities. Naively, we would expect the network to be symmetrical, since the properties of both modalities are the same. However, this behavior shows that other extrema of the objective function may exist, extrema which do not preserve the symmetry between the modalities. The simulation in Fig 7E serves as another example of how high plasticity can lead to synaesthesia, when comparing it to the simulation in Fig 7B. Again both had the same input statistics but different plasticity levels. It also demonstrates how sensory deprivation can lead to synaesthesia when comparing it to the simulation in Fig 7C. The simulations in Fig 7C and 7E had the same learning rate, but the magnitude of the inputs to modality 1 was reduced in the simulation of Fig 7E, resulting in a clear monotonic mapping (Fig 7E, right panel).

The high-dimensional model produces synaesthesia-like behaviour in response to the same kinds of parameter changes identified in the simple model: namely an increase in learning rate (analogous to high plasticity) and if one modality becomes more or less sensitive to its direct input relative to the other (sensory deprivation/flooding). This model also enabled us to explore the relationship between the inducer and concurrent. Although there was no correlated input during learning, the relationship between the inducer and concurrent tended to be monotonic, as is found in many naturally occurring forms of synaesthesia. This is not a trivial outcome, and suggests that such mappings are an emergent property of this kind of neural architecture.

## Discussion

For the last twenty years, theories of synaesthesia have been dominated by two general models: disinhibited feedback from multi-sensory regions to uni-sensory regions, and cross-talk theories which have emphasised the presence of atypical (and direct) structural connectivity between modalities [33]. Whereas the former explanation has tended to be favoured for explaining acquired synaesthesia, the latter has dominated explanations of developmental synaesthesia. The approach taken in our computational model represents a significant departure from this current status quo, and has generated novel insights. Our model repositions synaesthesia not as some quirk of aberrant connectivity but rather as a functional brain state that emerges, under certain conditions, as a consequence of optimising sensory information processing. In short, this model goes beyond others by offering an account not only of how synaesthesia emerges but also of why synaesthesia emerges. It offers a unifying account of acquired and developmental forms of synaesthesia insofar as it explains how the same outcome can emerge under different conditions within the same model.

Acquired synaesthesia is often associated with sensory deprivation due to damage to the sensory organs or pathways. Our model proposes that the same learning process that optimizes information representation naturally causes neurons in the deprived modality to enhance incoming inputs from intact modalities, leading to synaesthesia. To provide some intuition, we note that our model maximizes the output entropy of the network, which depends on two factors: one is the entropy of each single neuron, i.e. how variable the activity of single neurons is, and the other is the correlations among the neurons. Maximizing this entropy favours high single neuron entropy and low correlations among the neurons. The cross-talk connections induce correlations between the two modalities, which in general tend to reduce the output entropy. However, when one modality is deprived of input, it may be beneficial to have cross-talk connections from the intact modality to the deprived modality. The increase in the single neuron entropy due to the cross-talk connections can compensate for the higher correlations and result in a total increase of the output entropy. Loosely speaking, the deprived neurons seek for other neuronal sources of variability and enhance their connections with them. This mechanism, which emerges naturally in our computational framework, can also be useful for modelling the changes in neural representation that take place in other conditions such as phantom-limb [34].

Although functional accounts for acquired synaesthesia have been proposed in the past, no such comparable account has been put forward for developmental synaesthesia. Our model suggests that it arises from instability in the learning process due to high plasticity. It implies that synaesthetes have higher plasticity compared to non-synaesthetes or a relatively prolonged period of high-plasticity during childhood. Later on, as plasticity in the relevant brain areas decreases, the evolved cross-talk connections become stable. In line with this idea, whole-genome studies link some forms of synaesthesia to genes involved in plasticity, which have higher expression during early childhood [35]. Furthermore, developmental synaesthesia does not appear to be linked to sensory impairments and, if anything, is linked to increased perceptual sensitivities (notably within the concurrent modality). For instance, grapheme-colour synaesthetes show enhanced colour discrimination abilities [36]. In the proposed model, the recurrent connections within the concurrent modality amplify both its direct inputs and the ones from the inducer modality. Thus, an association between synaesthesia and increased perceptual sensitivity is an emergent property of the model, at least under certain scenarios, and it is important to explore the extent to which the presence of synaesthesia (cross-modal sensitivity) necessarily goes hand-in-hand with changes in intra-modal sensitivity. In terms of the underlying neurobiological mechanisms, the increased amplification by the recurrent interactions in our model is consistent with findings that indicate increased excitability and elevated glutamate concentration in the relevant cortical areas in synaesthetes [37, 38].

Traditionally, synaesthesia has not been linked to theories of learning and memory because it has been considered to reflect an innate (in its developmental form) cross-wiring of the senses. This view has been challenged on several fronts [e.g. 39, 40]. Firstly, many of the stimuli that induce synaesthesia (e.g. graphemes) are themselves learned. Secondly, for some synaesthetes the particular associations have been influenced by childhood coloured letter sets [13]. Moreover, some general cross-modal correspondences (e.g. between pitch and vertical positions) thought to reflect innate vestiges of synaesthesia have been shown to occur as statistical regularities in the environment [41]. Finally, synaesthetes (at least for grapheme-colour synaesthesia) are known to have better acquisition of new memories, and this may be related to increased plasticity during learning [32]. Future simulations of the model could use partially correlated inputs to the two modalities to model childhood exposure to coloured letter sets (they are not fully correlated given that most literacy exposure is with achromatic letters). It may well be the case that there is an interaction between learning rate (an innate parameter within the synaesthete brain) and these partial associations (in the environment), which explains why most people do not go on to develop synaesthesia after exposure to these stimuli.

An interesting hypothesis that emerges from this study regards the relationship between synaesthesia and the concept of critical brain dynamics [28, 42, 43]. The goal of the learning process in our model is to find the pattern of recurrent interactions that maximizes the sensitivity of the network to changes in its external inputs. In analogy to physical systems, in which the sensitivity (often termed susceptibility) to external inputs diverges near a critical point [44], here, as the network maximizes its sensitivity, it also tends to approach a critical point [28]. This critical point represents the border between normal amplification of external inputs and a regime governed by attractor dynamics. In the context of sensory processing, the super-critical attractor phase can be thought of as hallucinations that reflect the learned pattern of interactions. A useful measure for identifying critical dynamics is the time it takes the recurrent network to reach steady-state. When close to critical points, many dynamical systems display the phenomenon of *critical slowing down* [28, 45]. Interestingly, in simulations of the complex model in which synaesthesia evolved, when the learning process approached the optimal pattern of interactions, the dynamics of the recurrent network became substantially slower (the number of iterations required to process each input sample until reaching steady-state was ~35000–45000 compared to ~1000–4000 in the beginning of the learning process). This observation suggests that in the proposed model networks that developed synaesthesia operate closer to a critical point compared to networks that did not develop synaesthesia. The prediction is that there may be evidence of the neural signatures of critical dynamics in synaesthetes [46, 47], particularly as synaesthesia is developing.

In terms of its similarities to other models, our model resembles the direct cross-talk (or cross-activation) models proposed by others [48] primarily to account for developmental forms of synaesthesia. Although the model represents a direct form of cross-talk, it is an open question as to whether the model would produce similar patterns if neurons from modalities 1 and 2 were not directly connected but were themselves both connected via a third pool of neurons that receives no direct input from 1 and 2. There is some evidence for both direct and indirect types of neural architecture in synaesthesia as assessed via fMRI effective connectivity [49]. The addition of an interconnecting hub area in future modelling attempts would give the model top-down representations that could be adapted to the (Bayesian) predictive coding framework. Unlike the present (bottom-up) model, the predictive coding approach describes perception as top-down inference that is constrained and altered by sensory signals. A non-computationally explicit account of synaesthesia in terms of predictive coding has been articulated [50]. Moreover, the kinds of learning algorithms employed in our model are compatible with this approach [51].

The gradient-based learning rules used in this study are not local and are thus expected to reflect the long-term evolution of the system rather than mimicking the moment-by-moment dynamics of real neural circuits. In addition, the neurons in the model are described by simplified rate dynamics which do not capture the complex dynamics of real neurons. An important direction for future modelling work would be the examination of more biologically realistic networks that also optimize information representation. The scenarios for the evolution of synaesthesia described in this study are very general and we believe that similar scenarios would appear also in more realistic networks.

In summary, these computational models permit new ways of thinking about synaesthesia both in terms of causal mechanisms and in terms of optimising perceptual function. It generates non-trivial outcomes (e.g. generating monotonic mappings not found in the input characteristics) and non-trivial predictions (e.g. relating to learning, unimodal perceptual sensitivity, hallucinatory tendencies).

## Materials and Methods

The full details of the network model and the derivation of the learning algorithm appear in [27]. Here we briefly review the main ingredients of the model. The numerical simulations were performed in MATLAB. Analytical results for the simple model appear in S1 Appendix.

### General Network Architecture and Dynamics

The general architecture of the model is described in Fig 1. It involves an input layer with *N* neurons and an output layer with *M* neurons. We consider here only overcomplete representations, in which *M* ≥ *N*. In the simple model *M* = *N* = 2, and in the more complex model *N* = 4 and *M* = 142. The feedforward interactions are described by the *M x N* matrix *W* and the recurrent interactions by the *M x M* matrix *K*. During the presentation of each input sample, the input components *x*_*i*_ are fixed. The output neurons obey the following dynamics

$$\tau \frac{ds_{i}}{dt}=-s_{i}+g\left(\sum_{j=1}^{N}w_{ij}x_{j}+\sum_{k=1}^{M}k_{ik}s_{k}\right),\;\;\;\;i=1,\ldots ,M.$$

where *g* is some nonlinear squashing function and *τ* is a characteristic time scale. The steady-state responses are given by

$$s_{i}=g(\sum_{j=1}^{N}w_{ij}x_{j}+\sum_{k=1}^{M}k_{ik}s_{k}),\;\;\;\;i=1,\ldots ,M.$$

### Objective Function and Learning Algorithm

The representation of the external inputs is evaluated using the mutual information between the input and the steady-state output of the network [52]. The mutual information can be expressed as the difference between the entropy of the output and the conditional entropy of the output given the input. The conditional entropy represents the entropy of the output noise. Because the network response is a deterministic function of the input, the mutual information is functionally only dependent on the entropy of the outputs. As shown in [27], maximizing the output entropy (and therefore the mutual information) is equivalent to minimizing the following objective function:

$$\varepsilon =-\frac{1}{2}{\langle \text{ln}\;\text{det}\left(\chi^{T}\chi \right)\rangle }_{x}=-\frac{1}{2}Tr{\langle \text{ln}(\chi^{T}\chi )\rangle }_{x},$$

where $\chi_{ij}=\frac{\partial s_{i}}{\partial x_{j}}$ is the Jacobian matrix of the transformation and reflects the sensitivity of the output units to changes in the input units. We also refer to this matrix as the susceptibility matrix as it is analogous to the susceptibility of physical systems to external fields.

The adaptive parameters of the algorithm are the sets of feedforward and recurrent interactions, *W*_*ij*_ and *K*_*ij*_. The learning rules for these parameters are derived from this objective function using the gradient decent method, as shown in [27]. Here we focus only on the recurrent interactions. The gradient descent learning rule for the recurrent interactions is

$$\Delta K=-\eta \frac{\partial \varepsilon }{\partial K}=\eta \langle {\left(\chi \Gamma \right)}^{T}+\phi^{T}as^{T}\rangle ,$$

where *η* is the learning rate, the matrix *ϕ* is given by *ϕ* = (*G*^−1^−*K*)^−1^ and satisfies *χ* = *ϕW*, the matrix *G* is defined as *G*_*ij*_ = *g*′_*i*_ *δ*_*ij*_, the matrix Γ is defined as Γ = (*χ*^*T*^*χ*)^−1^*χ*^*T*^*ϕ* and the components of the vector *a* are given by $a_{k}={\left[\chi \Gamma \right]}_{kk}\frac{{g^{\prime \prime }}_{k}}{{\left({g^{\prime }}_{k}\right)}^{3}}$. The triangular brackets denote averaging over the input samples.

During the learning process, the evolving networks can approach a critical point in their dynamics (see Discussion). In such cases, the objective function becomes very sensitive to changes in the pattern of interactions. In some cases the objective function may even increase rather than decrease. One way to avoid this is to gradually reduce the learning rate to very small magnitudes. However, to minimize the number of free parameters and make the interpretation clearer, we chose to leave the learning rate fixed across the learning process. Rather, we saved the interaction patterns in the course of the learning process and if a substantial increase in the objective function was identified, we simply chose the interaction pattern associated with the minimal value of the objective function, namely the optimal pattern. To estimate the convergence time of the recurrent network and identify critical slowing down, we defined a criterion for stability of the neuronal activities and measured the time it takes the network to satisfy this criterion. A substantial increase in the convergence time suggests that the network operates close to a critical point. We indeed observed such substantial slowdown of the network dynamics, in particular in the simulations that developed synaesthesia when they approached the optimal pattern of interactions. As a consequence, the simulations could be very long (up to a couple of weeks on a standard PC station).

## Supporting Information

S1 Appendix Analytical derivation of the conditions for the evolution of cross-talk in the simple model. (DOCX)
