# Supplementary material for: The Emergence of Synaesthesia in a Neuronal Network Model via Changes in Perceptual Sensitivity and Plasticity
Source: PLoS Comput Biol. 2016 Jul 8;12(7):e1004959. doi: 10.1371/journal.pcbi.1004959 (PMC4938560; doi:10.1371/journal.pcbi.1004959)
Supplement: S1 Appendix — (DOCX) [file pcbi.1004959.s001.docx]

**Appendix**

**Analytical derivation of the conditions for the evolution of cross-talk in the simple model**

The activities of the input neurons (*x*) and the output neurons (*s*) are represented by the two-dimensional vectors:

$x=\left[\begin{matrix} x_{1} \\ x_{2} \end{matrix}\right]$ , $s=\left[\begin{matrix} s_{1} \\ s_{2} \end{matrix}\right]$

When the network is presented with an input sample, the neuronal activities change in time according to the following first order dynamics:

$\tau\frac{ds_{1}}{dt}=-s_{1}+g\left(w_{1}x_{1}+k_{12}s_{2}\right) , \tau\frac{ds_{2}}{dt}=-s_{2}+g\left(w_{2}x_{2}+k_{21}s_{1}\right)$,

where$\tau$is a time constant. After the recurrent network reaches steady-state, the activities are given by:

$$s_{1}=g\left(w_{1}x_{1}+k_{12}s_{2}\right)\equiv g\left(h_{1}\right) , s_{2}=g\left(w_{2}x_{2}+k_{21}s_{1}\right)\equiv g\left(h_{2}\right)$$

The function g is a non-linear monotonic (invertible) function.

The recurrent interactions form a 2x2 matrix, *K*. Assuming no self-coupling, the main diagonal is set to zero:

$$K=\left[\begin{matrix} 0 & k_{12} \\ k_{21} & 0 \end{matrix}\right]$$

The information maximization learning rule for the recurrent interactions in this case is (Methods; 27):

$$\Delta k=\eta\left\langle\varphi^{T}+\varphi^{T}as^{T}\right\rangle$$

where $\eta$ is the learning rate. The matrix $\varphi$ is given by: $\varphi=\left(I-GK\right)^{-1}G=\left(G^{-1}-K\right)^{-1}$,

where G is a diagonal matrix with elements $G_{ij}=g'(h_{i})\delta_{ij}\equiv g'_{i}\delta_{ij}$.

The components of the vector *a* are given by:

The triangular brackets denote averaging over the input samples. Applying this to our simple network model, we obtain the following equations for the off-diagonal elements of the interaction matrix:

$$\Delta k_{12}=\eta\left\langle q\cdot g'_{1}g'_{2}k_{21}+q^{2}\cdot\left(\frac{g''_{1}}{g'_{1}}+\frac{g'_{1}g''_{2}}{g'_{2}}k_{21}\right)s_{2}\right\rangle\equiv\eta\left\langle f_{1}\right\rangle$$

$$\Delta k_{21}=\eta\left\langle q\cdot g'_{1}g'_{2}k_{12}+q^{2}\cdot\left(\frac{g''_{2}}{g'_{2}}+\frac{g'_{2}g''_{1}}{g'_{1}}k_{12}\right)s_{1}\right\rangle\equiv\eta\left\langle f_{2}\right\rangle$$

where .

The resulting learning rules form a set of two coupled dynamical equations of the form:

$$\left[\begin{matrix} \Delta k_{12} \\ \Delta k_{21} \end{matrix}\right]=\eta\left\langle\left[\begin{matrix} f_{1}\left(k_{12},k_{21}\right) \\ f_{2}\left(k_{12},k_{21}\right) \end{matrix}\right]\right\rangle$$

We assume that under normal conditions, when there are no correlations between the inputs to the two modalities, a state of no cross-talk should be a fixed-point of the learning dynamics. The conditions for this state to be a fixed-point are:

$$\left.\left[\begin{matrix} \Delta k_{12} \\ \Delta k_{21} \end{matrix}\right]\right|_{K=0}=0$$

Non-zero cross-talk connections will evolve when this fixed point becomes unstable.

In order to analyze the behavior of the learning dynamics near a state of no cross-talk, we linearize the learning rules around the fixed point of *K*=0:

Where:

,

, $\left.\frac{\partial f_{2}}{\partial k_{12}}\right|_{K=0}=\left.\frac{\partial f_{1}}{\partial k_{21}}\right|_{K=0}$

The discrete-time dynamics are given by:

$$\left[\begin{matrix} k_{12} \\ k_{21} \end{matrix}\right]_{n+1}=\left[\begin{matrix} k_{12} \\ k_{21} \end{matrix}\right]_{n}+\eta J\left[\begin{matrix} k_{12} \\ k_{21} \end{matrix}\right]_{n}=\left(I+\eta J\right)\left[\begin{matrix} k_{12} \\ k_{21} \end{matrix}\right]_{n}\equiv A\left[\begin{matrix} k_{12} \\ k_{21} \end{matrix}\right]_{n}$$

and the formal solution is:

$$\left[\begin{matrix} k_{12} \\ k_{21} \end{matrix}\right]_{n}=A^{n}\left[\begin{matrix} k_{12} \\ k_{21} \end{matrix}\right]_{0}$$

The condition for stability is convergence of$A^{n}$ when $n\to\infty$. Therefore, the eigenvalues of *A* must satisfy:

$$\left|\gamma_{i}\right|<1$$

The eigenvalues of *A* can be expressed using those of J:

$$\gamma_{i}=1+\eta\cdot\lambda_{i}$$

The eigenvalues of the matrix *J* are:

$$\lambda_{1,2}=\frac{1}{2}\cdot\left[Tr\left(J\right)\pm\sqrt{Tr\left(J\right)^{2}-4Det\left(J\right)}\right]$$

Where:

To be concrete, we choose the logistic function:

$$g\left(x\right)=\frac{1}{1+e^{-x}}$$

This function satisfies the following relations:

We obtain:

Using symmetry considerations, we also obtain:

$\left.\frac{\partial f_{2}}{\partial k_{21}}\right|_{K=0}=-2s_{2}s_{1}^{2}+2s_{1}^{2}s_{2}^{2}$ , $\left.\frac{\partial f_{2}}{\partial k_{12}}\right|_{K=0}=\left.\frac{\partial f_{1}}{\partial k_{21}}\right|_{K=0}$

The trace and determinant are thus given by:

$$\tau\equiv Tr\left(J\right)=4\left\langle s_{1}^{2}s_{2}^{2}\right\rangle-2\left\langle s_{1}s_{2}^{2}\right\rangle-2\left\langle s_{2}s_{1}^{2}\right\rangle$$

$$\Delta\equiv Det\left(J\right)=4\left(\left\langle s_{1}^{2}s_{2}^{2}\right\rangle-\left\langle s_{1}s_{2}^{2}\right\rangle\right)\cdot\left(\left\langle s_{1}^{2}s_{2}^{2}\right\rangle-\left\langle s_{2}s^{2}_{1}\right\rangle\right)-\left(3\left\langle s_{1}s_{2}\right\rangle-4\left\langle s_{1}s_{2}^{2}\right\rangle-4\left\langle s_{2}s_{1}^{2}\right\rangle+5\left\langle s_{1}^{2}s_{2}^{2}\right\rangle\right)^{2}$$

We assume that $s_{1}$ and $s_{2}$ are statistically independent. Therefore:

$$\left\langle s_{1}^{n}s_{2}^{m}\right\rangle=\left\langle s_{1}^{n}\right\rangle\left\langle s_{2}^{m}\right\rangle$$

The requirement for *K*=0 to be a fixed point of the learning dynamics gives the following condition:

The only relevant solution is:

$$\left\langle s_{1}\right\rangle=\left\langle s_{2}\right\rangle=\frac{1}{2}$$

Denoting $\left\langle s_{i}^{2}\right\rangle=\alpha_{i}^{2}$ we obtain

$$\tau=4\alpha_{1}^{2}\alpha_{2}^{2}-\alpha_{1}^{2}-\alpha_{2}^{2}$$

$$\Delta=-\frac{9}{16}+3\alpha_{1}^{2}+3\alpha_{2}^{2}-4\alpha_{1}^{4}-4\alpha_{2}^{4}-\frac{29}{2}\alpha_{1}^{2}\alpha_{2}^{2}+18\alpha_{1}^{2}\alpha_{2}^{4}+18\alpha_{1}^{4}\alpha_{2}^{2}-21\alpha_{1}^{4}\alpha_{2}^{4}$$

The eigenvalues of the matrix *A* should satisfy:

$$\left|\gamma_{1,2}\right|=\left|1+\frac{1}{2}\eta\cdot\left(\tau\pm\sqrt{\tau^{2}-4\Delta}\right)\right|<1$$

Thus, the following inequalities must be satisfied:

$\tau<0$,$\Delta>0$ (else we get$\gamma_{1}>1$).

There are two options that we need to consider – real eigenvalues and complex. If the eigenvalues are complex, their magnitudes are:

$$\left|\gamma_{1,2}\right|=\left|1+\frac{1}{2}\eta\cdot\left(\tau\pm\sqrt{\tau^{2}-4\Delta}\right)\right|=\sqrt{\left(1+\frac{1}{2}\eta\tau\right)^{2}+\left(\frac{1}{2}\eta\sqrt{4\Delta-\tau^{2}}\right)^{2}}=\sqrt{1+\eta\tau+\eta^{2}\Delta}$$

The critical value of $\eta$is when the magnitude equals 1:

$$\sqrt{1+\eta\tau+\eta^{2}\Delta}=1\Rightarrow\eta\tau+\eta^{2}\Delta=0 \Rightarrow\eta=-\frac{\tau}{\Delta}$$

If the eigenvalues are real, we have to check that$\lambda_{1}$ (the larger eigenvalue) is no greater than 1 and that $\lambda_{2}$ (the smaller eigenvalue) is no smaller than -1:

$$1+\frac{1}{2}\eta_{critical}\cdot\left(\tau+\sqrt{\tau^{2}-4\Delta}\right)=1 \Rightarrow\frac{1}{2}\eta_{c}\cdot\left(\tau+\sqrt{\tau^{2}-4\Delta}\right)=0\Rightarrow\tau+\sqrt{\tau^{2}-4\Delta}=0$$

Thus,$\gamma_{1}$does not depend on $\eta$.

$$1+\frac{1}{2}\eta_{critical}\cdot\left(\tau-\sqrt{\tau^{2}-4\Delta}\right)=-1\Rightarrow\frac{1}{2}\eta_{c}\cdot\left(\tau-\sqrt{\tau^{2}-4\Delta}\right)=-2\Rightarrow\eta_{c}=-\frac{\tau+\sqrt{\tau^{2}-4\Delta}}{\Delta}$$

The value of $\eta_{c}$can be written in general (for both complex and real eigenvalues) as:

$$\eta_{c}=-\frac{\tau}{\Delta}+\mathrm{Re}\left(\frac{\sqrt{\tau^{2}-4\Delta}}{\Delta}\right)$$

Numeric calculations show that under the conditions of $\tau<0$,$\Delta>0$ the discriminant is positive. Therefore the relevant solution is $\eta_{c}=-\frac{\tau+\sqrt{\tau^{2}-4\Delta}}{\Delta}$. Note that when $\alpha_{1},\alpha_{2}\to\infty$, this expression diverges to infinity.

As shown above, the value of $\gamma_{1}$ does not depend on $\eta$, but does depend on $\alpha_{1}^{2},\alpha_{2}^{2}$. It can be noticed that the only solution for $\tau+\sqrt{\tau^{2}-4\Delta}=0$is when$\Delta=0$. In this case, $\gamma_{1}$reaches the critical value of 1, and the dynamics become unstable. The condition for this instability is:

$$\begin{matrix} \Delta=-\frac{9}{16}+3\alpha_{1}^{2}+3\alpha_{2}^{2}-4\alpha_{1}^{4}-4\alpha_{2}^{4}-\frac{29}{2}\alpha_{1}^{2}\alpha_{2}^{2}+18\alpha_{1}^{2}\alpha_{2}^{4}+18\alpha_{1}^{4}\alpha_{2}^{2}-21\alpha_{1}^{4}\alpha_{2}^{4}=0 \\ \Rightarrow\alpha_{1}^{2}=\frac{36\alpha_{2}^{4}-29\alpha_{2}^{2}+6\pm\sqrt{-6\alpha_{2}^{2}\left(2\alpha_{2}^{2}-1\right)^{3}}}{84\alpha_{2}^{4}-72\alpha_{2}^{2}+16} \end{matrix}$$

This relation defines the curves in Figures 3A, 3B and 4.

The expression in the root cannot be negative, therefore$0\leq\alpha_{2}^{2}\leq\frac{1}{2}$. From symmetry considerations we also obtain$0<\alpha_{1}^{2}<\frac{1}{2}$.

The variance of the output neuron's activity is:

$\sigma_{i}^{2}=\left\langle\left(s_{i}-\left\langle s_{i}\right\rangle\right)^{2}\right\rangle=\left\langle s_{i}^{2}-2s_{i}\left\langle s_{i}\right\rangle+\left\langle s_{i}\right\rangle^{2}\right\rangle=\left\langle s_{i}^{2}\right\rangle-2\left\langle s_{i}\right\rangle^{2}+\left\langle s_{i}\right\rangle^{2}=\left\langle s_{i}^{2}\right\rangle-\left\langle s_{i}\right\rangle^{2}=\alpha_{i}^{2}-\frac{1}{4}\leq\frac{1}{2}-\frac{1}{4}\leq\frac{1}{4}$Thus: $\frac{1}{4}\leq\alpha_{i}^{2}\leq\frac{1}{2}$ or $0\leq\sigma_{i}^{2}\leq\frac{1}{4}$
